# *Saccharomyces*IDentifier, *S*ID: strain-level analysis of *Saccharomyces cerevisia*e populations by using microsatellite meta-patterns

**DOI:** 10.1038/s41598-017-15729-3

**Published:** 2017-11-10

**Authors:** Irene Stefanini, Davide Albanese, Maddalena Sordo, Jean-Luc Legras, Carlotta De Filippo, Duccio Cavalieri, Claudio Donati

**Affiliations:** 1 Computational Biology Unit, Fondazione Edmund Mach, via E. Mach 1, 38010 San Michele all’Adige, Trento, Italy; 2 UMR 1083 INRA, Montpellier-Supagro, Université Montpellier 12 place Viala, 34061 Montpellier Cedex 1, France; 3 Institute of Agricultural Biology and Biotechnology, National Research Council (CNR), Pisa, Italy; 4 Present Address: Division of Biomedical Cell Biology, University of Warwick, Coventry, CV4 7AL United Kingdom; 5 Present Address: Department of Biology, University of Florence, via della Madonna del Piano 6, 50019 Sesto Fiorentino, Florence Italy

## Abstract

*Saccharomyces cerevisiae* is a common yeast with several applications, among which the most ancient is winemaking. Because individuals belonging to this species show a wide genetic and phenotypic variability, the possibility to identify the strains driving fermentation is pivotal when aiming at stable and palatable products. Metagenomic sequencing is increasingly used to decipher the fungal populations present in complex samples such as musts. However, it does not provide information at the strain level. Microsatellites are commonly used to describe the genotype of single strains. Here we developed a population-level microsatellite profiling approach, *S*ID (*Saccharomyces cerevisiae* IDentifier), to identify the strains present in complex environmental samples. We optimized and assessed the performances of the analytical procedure on patterns generated *in silico* by computationally pooling *Saccharomyces cerevisiae* microsatellite profiles, and on samples obtained by pooling DNA of different strains, proving its ability to characterize real samples of grape wine fermentations. *S*ID showed clear differences among *S*. *cerevisiae* populations in grape fermentation samples, identifying strains that are likely composing the populations and highlighting the impact of the inoculation of selected exogenous strains on natural strains. This tool can be successfully exploited to identify *S*. *cerevisiae* strains in any kind of complex samples.

## Introduction

In the metagenomic era, Next Generation Sequencing allows the characterization of the composition and dynamics of the complex microbial communities present in almost every kind of sample. While allowing us to obtain a general picture of the microbiota, amplicon-based approaches have a taxonomic resolution that usually does not exceed the genus or, in the best situations, the species level^1^. Although this is in general sufficient, in some situations a higher taxonomic resolution is necessary. As an example, in late stages of the wine fermentation process when the ethanol concentration exceeds the tolerable level for the majority of bacteria and environmental fungi, the microbial population simplifies and is usually dominated by the budding yeast *Saccharomyces cerevisiae* ^2,3^. Recent surveys have shown how the wide phenotypic variability of *S*. *cerevisiae* impacts its ability to ferment grape must and produce metabolites relevant for the organoleptic characteristics of the fermented product^4–6^. Hence, winemakers usually inoculate the fresh must with selected *S*. *cerevisiae* strains to overgrow the natural microbial populations (potentially responsible of spoilage) and to guarantee the final product a specific organoleptic profile^7^. Hundreds of different strains are nowadays available to conduct different grape types fermentations^8^. However, several studies have shown that in some cases environmental strains can overgrow inoculated strains^9–15^. In these cases, it has been suggested that the role of the inoculum of alien *S*. *cerevisiae* strains is to facilitate the growth of indigenous *S*. *cerevisiae* strains (fitter than the alien ones when in competition) by setting up a hostile environment for other fungal species^16^. This situation represents a golden opportunity because of the renewed interest in the use of indigenous yeasts among winemakers and scientists in the last decades^17^. In this case, a strain unable to compete with the indigenous *S*. *cerevisiae* strains would be preferred to strains able to overgrow the natural fungal population. The development of rapid methods characterizing mixed populations composed by multiple *S*. *cerevisiae* strains will be instrumental to evaluate the performance of selected strains inoculated in musts. There are other cases in which the ability to discriminate among *S*. *cerevisiae* strains holds great potentials. Despite being generally considered a commensal, several recent studies have reported the emergence of *S*. *cerevisiae* as an opportunistic pathogen^18–20^. *S*. *cerevisiae* was enriched in the gut mucosa of Crohn disease patients, thus suggesting a negative relation between the abundance of this yeast and the health status of the host^21^. Conversely, a reduction of this yeast was found in the feces of Crohn patients in disease versus remission, thus proposing a positive role of *S*. *cerevisiae* colonization^22^. These controversial results led to the hypothesis that, rather than the presence of this yeast, the presence of different *S*. *cerevisiae* strains could have different effects on the host health status^23^. Another interesting environment in which several *S*. *cerevisiae* strains have been found is the insect intestines^24,25^. Even in this case, the effect on the host health is still debated. Beekeepers are aware of the positive effect of *S*. *cerevisiae* on the insect, in fact they usually feed the bees with the baker’s yeast after the winter or after the harvesting to let the colony recover. However, reports indicate that stressed bees show higher amounts of yeasts than usual, but it is not clear whether this is a consequence or a cause of the stress^26^. In all these situations, the ability to rapidly identify the *S*. *cerevisiae* strains present in the complex matrix could help understanding the role of different strains in the host health. Despite the acknowledged necessity to assess the variability of *S*. *cerevisiae* populations, the identification of their composition has been so far mainly carried out by means of isolation, a process requiring time and specialized operators^9,12–15^. Only a few molecular approaches have been proposed, but these are aimed at the dissection of the complete fungal population, rather than focusing on *S*. *cerevisiae* ^2^. Simple Sequence Repeats (SSRs), also called microsatellites, are non-coding DNA sequences composed by small repeated units (2–6 bp). The number of small units repetitions varies in different individuals, making thus SSRs good markers for the high resolution typing of individuals^27^. Since unrelated individual strains can harbor the same SSR allele in a given locus, several different microsatellite loci are usually combined for the typing of an individual^28^. SSRs-based approaches have been successfully applied in population genetic studies to characterize the microevolution and environmental distribution of *S*. *cerevisiae* isolates^29,30^. Until now, all genotyping analyses have been performed on individual strains. However, in several cases (e.g. maize^31^, humans^32^), genotyping of pools of individuals has been used as a tool for the comparison of populations. Recently, a multiplex PCR-SSR analysis was proposed to monitor inoculated yeast strains in industrial wine fermentation^33^. By applying this technique, the authors were able to compare the band profiles (on agarose gels) of different samples and to draw conclusions on the persistence of inoculated strains. Nevertheless, the resolution of this analysis did not allow the identification of the different strains present in the complex samples^33^.

In this work we propose a technology based on SSRs analysis to characterize complex blends of *S*. *cerevisiae* strains. This new approach allows the rapid and exhaustive investigation of different *S*. *cerevisiae* populations at the strain level by evaluating which combination of strains, chosen from a representative reference dataset, is present in the given complex sample. We have developed a new open-access tool, *S*ID (*Saccharomyces cerevisiae* IDentifier, https://sidentifier.shinyapps.io/SIDentifier/), through which specialized and not-specialized workers (i.e. wine-makers) could easily characterize the *S*. *cerevisiae* strains driving fermentations.

## Results

With the SSR meta-profiling we set up a procedure to identify the strains composing mixed samples. As a starting point, we assessed the performance of twelve microsatellites loci in describing synthetically generated *S*. *cerevisiae* pools. We then used these pools to test the ability of GLM (Generalized Linear Model) analysis to identify the parental strains. We further evaluated lasso^34^ (least absolute shrinkage and selection operator) analysis performances on a large number of samples by analysing a dataset generated *in silico* and composed by randomly combined single strains profiles. We finally tested the method on real samples of fermenting grape musts.

### Evaluation of the SSRs meta-profiling performance on pooled strains

**Figure 1:**
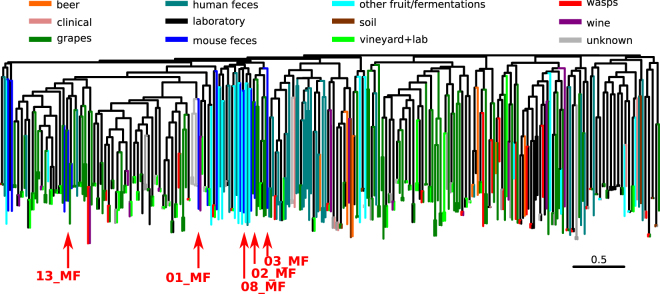
Neighbor-joining tree based on the Dc chord distances calculated on microsatellite data of a collection of Saccharomyces cerevisiae isolates. The strains selected for the synthetic pooling are highlighted by arrows.

We generated a set of synthetic pools by mixing the DNAs of selected strains. To select the strains to be pooled, we initially assessed the genotype of 292 environmental and laboratory strains by mean of SSR analysis (Fig. 1). Twelve microsatellite loci were analyzed: C3, C4, C5, C6, C8, C11, SCYOR267c, SCAAT1, SCAAT3, SCAAT5, YKL172W and YPL9. We then built a neighbor-joining tree using the Dc chord distance matrix calculated on the microsatellites data (Fig. 1). The strains to be pooled were selected using the following criteria: *i*) they should be preferentially isolated from the same source (to mimic the real application of the method), *ii*) the pool should encompass both genetically similar and different strains (to assess the discrimination ability -D_a_- of the method). With these criteria in mind, we finally selected five *S*. *cerevisiae* strains isolated from faeces and with variable degree of differentiation among each other (Fig. 1). To note, the strains 02_MF and 04_MF were almost identical (0.069 Dc-chord distance), while 08_MF was the most dissimilar from 01_MF and 13_MF (0.809 and 0.654 Dc-chord distance, respectively). We included highly similar strains to test the performance of the method in the most difficult settings, where two almost genetically identical strains present in the same sample have to be recognised as different. The pools were generated as described in methods by combining from two to five “parental” strain DNAs (Supplementary Table S1). The same twelve microsatellites loci used to select the strains were also amplified in the synthetic pools. The profiles obtained for the pooled DNAs (pooled profiles) were compared to the expected profiles composed by all the alleles present in the parental strains. The performance of each locus was evaluated by calculating the average error (E_a_) among the samples (see equation 2). The C3, YOR267c, SCAAT5 and YKL172 SSR loci performed excellently, with all the alleles of the parental strains being identified in all the tested samples (corresponding to E_a_ = 0 in Table 1). On the contrary, the C6 and C8 loci showed a high E_a_, 8.77% and 4.63% (Table 1). These loci showing bad performances when considered separately might affect the ability of our proposed method in discriminating the strains present in complex samples. For this reason, in the following analyses we assessed the performances of two different sets of loci: “full” - encompassing all the 12 loci-, and “reduced” - in which the C6 and C8 loci were excluded-. The profiles of the parental strains were inspected aiming at the identification of strain-specific alleles, namely the alleles present in only one of the pooled strains. Discrimination ability (D_a_) was calculated as the number of strains that could be identified by an allele found in the pool on the total of pooled strains (see equation 1). Despite the fact that this index is related to the set of pooled samples (none of the reference collection strains bears a specific allele when compared to the whole collection), it is useful to evaluate the contribution of each locus in discriminating the different pooled strains. In general, the D_a_ of all the SSR loci decreased with the increase of the number of pooled strains, due to the fact that the same allele can be present in more than one different strain. Indeed, given their genetic similarity (Supplementary Figure S1), the selected strains are probably the result of a clonal expansion of a strain in the same isolation source, or result from inbreeding of closely related strains as shown in other isolation sources^35^. For these reasons, profiling of the alleles at several different microsatellite loci is needed to genetically characterize *S*. *cerevisiae* isolates.

**Table 1:** Performances of the tested SSR loci.

Locus ID	E_a_	D_a_					
		A^1^	B^1^	C^1^	D^1^	E^1^	F^1^
C3	0.00	1/2	0/2	1/3	1/3	1/4	1/5
C4	0.69	1/2	0/2	2/3	2/3	2/4	2/5
C5	0.45	2/2	0/2	2/3	2/3	3/4	3/5
C6	8.77	1/2	0/2	2/3	2/3	2/4	2/5
C8	4.63	0/2	1/2	0/3	1/3	1/4	1/5
C11	0.57	1/2	0/2	1/3	1/3	1/4	1/5
YOR267c	0.00	2/2	1/2	3/3	2/3	3/4	3/5
SCAAT1	0.20	1/2	0/2	2/3	2/3	2/4	2/5
SCAAT3	0.64	1/2	0/2	2/3	2/3	2/4	2/5
SCAAT5	0.00	0/2	0/2	0/3	0/3	0/4	0/5
YPL9	0.607	1/2	0/2	2/3	2/3	2/4	2/5
YKL172	0.00	1/2	0/2	1/3	1/3	1/4	1/5

Average locus error E_a_ was calculated as the fraction of alleles of the given locus in the query sample not matching the combination of the identified strains profiles divided by the number of alleles for the given locus (see equation 2); D_a_ = discrimination ability = number of pooled strains which were identified by at least 1 characteristic allele divided by the total number of pooled strains, see equation 1. ^1^Labels refer to the synthetic pool name, as listed in Supplementary Table 1.

### Test for strain identification - pooled strains

The SSR meta-profiling approach allows the identification of the strains composing complex populations of *S*. *cerevisiae*. Indeed, as the SSR alleles at different loci allow the identification and genetic comparison of single strains^28^, we tested whether a penalized regression approach was able to disentangle the individuals composing a complex population. The performances of the method were evaluated on the synthetic patterns obtained by SSRs characterization of the mixed pools used in the previous section (Supplementary Table S1). The pooled strains samples showed from a minimum of 2 (in pools composed by two strains) up to 6 alleles (in pools composed by three or more strains) per locus. The GLM analysis exactly identified the parental patterns (True positive rate = 100%, equation 4), with the only exception of the 08_MF strain, which was not identified in the F sample (Table 2). In addition, the number of false positives was low (Table 2). One of the false positives identified (03_MF in pool A and C) was genetically very similar to the 02_MF strain present in the pools (Dc chord distance lower than 0.1, Supplementary Figure S1). To note, for the synthetic pool D containing the same strains as the C pool with the only difference of the 03_MF strain in spite of the 02_MF strain, the identification was correct.

**Table 2:** Parent profile identification from synthetic patterns.

Sample Name	Expected	Identified	True positive rate	False positives	GLMerror*
A	01_MF+02_MF	**01_MF, 02_MF**, 03_MF	100%	1	0.61
B	02_MF+03_MF	**02_MF, 03_MF**	100%	0	1.22
C	01_MF+02_MF+13_MF	**01_MF, 02_MF**, 03_MF, **13_MF**	100%	1	0.30
D	01_MF+03_MF+13_MF	**01_MF, 03_MF, 13_MF**	100%	0	0.30
E	01_MF+02_MF+03_MF+13_MF	**01_MF, 02_MF, 03_MF, 13_MF**	100%	0	0.30
F	01_MF+02_MF+03_MF+08_MF+13_MF	**01_MF, 02_MF, 03_MF, 13_MF**	80%	0	0.30

In the “Identified” column, the strains ID in bold are the parental strains correctly identified by the GLM. True positive rate is the percentage of parental strains identified in the sample by the model. The column “False positives” indicates the number of strains identified by lasso analysis but not present in the query sample. *GLMerror was estimated as the percentage of alleles differing between the query sample and the combination of the identified strains’ patterns, on the total of alleles (equation 3).

### Test for strain identification - in silico pools

**Figure 2:**
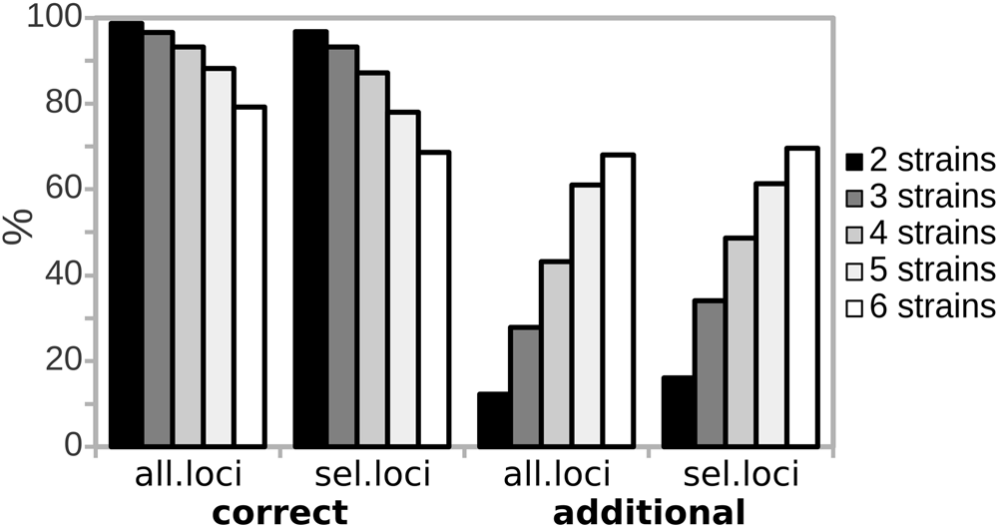
*Summary of the results of penalized GLM analysis on in silico*-generated complex profiles. *In silico* profiles were generated by randomly combining 2, 3, 4, 5 or 6 single-strain profiles (1000 profiles each), then analysed by mean of GLM. The y-axis reports the percentage of *in silico* samples for which either all the parental strains (left part of the plot) or additional strains/false positives (more than these actually combined to generate the complex profile, right part of the plot) were identified by GLM analysis.

To further evaluate the performance of the approach on a larger set of samples, we generated *in silico* patterns by randomly combining from 2 to 6 single strain SSR patterns from the reference strain collection (1000 patterns for each combination). As described in previous section, we evaluated the performance of two sets of SSRs loci: “all.loci” – encompassing all the 12 loci- and “sel.loci” - in which the C6 and C8 loci were excluded- (Table 1). GLM analysis was then applied to the *in silico* dataset, using as observations (reference) the patterns of the single individuals (the strains of the collection used in Fig. 1) and each *in silico* pattern as variable (query). For the pools composed by 2 profiles, the parental strains were correctly identified in the 98.7% of the cases using the full set of loci and in the 96.8% of the cases using the reduced set of loci (Fig. 2). The percentage of pools for which all the parental strains were correctly identified decreased with the increase of the number of profiles pooled together (Fig. 2), down to the 79.2% of the cases for the most complex *in silico* samples (composed by 6 strains). This percentage dropped to the 69.6% of the cases when using the reduced set of loci. Despite the previously observed bad performance of some SSR loci (Table 1), GLM analysis performed better when using the complete set of SSRs than when removing the problematic loci. We thus decided to use the whole SSR set in the following analyses.

The percentage of pools in which at least one false positive was identified increased with increasing sample complexity (Fig. 2). In fact, when considering all the microsatellite loci, in the 12.3% of the samples composed by 2 strains more than 2 strains were identified, and this rate increased up to the 68% for the samples composed by 6 strains. The results obtained on the *in silico* data indicate that the procedure allows to identify the patterns composing a given complex samples in the large majority of cases, but can over-estimate the real richness of complex samples.

### Real samples

As a proof of concept, we applied the proposed method to a set of spontaneous and inoculated wine must fermentations. The fermentations of several grape cultivars from the same cellar were studied: Traminer, Chardonnay, Muller-Thurgau, Solaris and Sauvignon. In addition, the fermentations of Sauvignon and Muller-Thurgau were carried out by either exploiting the natural microbiota or by the inoculation of selected starters (list of samples in Supplementary Table S2). To exploit the natural yeast population, Muller-Thurgau mature grapes were collected and pressed before the harvesting, and the obtained must was fermented at high temperatures (greater than 20 °C) to facilitate *S*. *cerevisiae* overgrowth. This process was carried out in the cellar and used as one of the inocula (indicated as “*pied de cuve*”). The fermentations of Sauvignon and Muller-Thurgau musts lasted between 8 and 10 days, with the Muller-Thurgau fermentations being the slowest (10 days). The fungal populations present in the samples were initially explored at the species level by mean of PCR-RFLP on the ITS1-5.8-ITS2 region. The patterns observed in fresh musts showed several bands of different lengths (Supplementary Figure S2), indicating the presence of different yeast species in these samples. Later, at the fourth day of fermentation, the SSR pattern became simpler in all the ferments, with a lower number of bands. The patterns observed since the fourth day of fermentation until the end of the process showed the typical band profile of *S*. *cerevisiae*, indicating that this species over-grew the rest of the fungal population already at this early stage. We applied the SSR meta-profiling approach to dissect the composition of the *S*. *cerevisiae* populations present in all the samples (even the earliest ones, showing several yeast species). The twelve microsatellite loci tested on the synthetic pools were characterized by mean of penalized GLM analysis. In fresh musts, both of Muller-Thurgau and Sauvignon, two strains were identified, closely related to 02_MF and 03_MF (Table 3). While evaluating the performances of the approach on pooled strains, we observed that, while the 03_MF strain can be correctly identified, the 02_MF strain can be identified as 02_MF, 03_MF or both (Table 2). Thus, the Muller-Thurgau and Sauvignon samples could be populated either by the 02_MF strain alone or by both the 02_MF and the 03_MF strains. In any case, the identification of the same strains in musts obtained from grapes of different cultivars could indicate a cellar origin of the found *S*. *cerevisiae* strains. The same two strains were also identified in all the samples inoculated with the *pied de cuve*, both being Muller-Thurgau and Sauvignon musts, indicating that the *S*. *cerevisiae* strains present in the fresh must persisted during the entire fermentation process. Similarly, in the other tested must types (Solaris, Traminer and Chardonnay) inoculated with *pied de cuve* the same two strains were identified, suggesting a cellar origin.

**Table 3:** Strains identified by *S*ID and delta amplification in must samples.

Sample	Must type	Inoculum	*SID* strains	GLMerror^1^	delta amplif. strains	cluster^2^
*Pied de cuve*	Muller-Thurgau	*Pied de cuve*	02_MF; 03_MF	3.08*	A	A = 02_MF/03_MF
					B	B = 02_MF/03_MF
					C	C = 02_MF/03_MF
MullerMust	Muller-Thurgau	none	02_MF; 03_MF	2.16	B	B = 02_MF/03_MF
					C	C = 02_MF/03_MF
Muller_Pdc_4dd	Muller-Thurgau	*Pied de cuve*	02_MF; 03_MF	2.16	A	A = 02_MF/03_MF
Muller_Pdc_8dd	Muller-Thurgau	*Pied de cuve*	02_MF; 03_MF	1.54	A	A = 02_MF/03_MF
					B	B = 02_MF/03_MF
Muller_Pdc_EF	Muller-Thurgau	*Pied de cuve*	02_MF; 03_MF	2.16	A	A = 02_MF/03_MF
					B	B = 02_MF/03_MF
Muller_prep1_4dd	Muller-Thurgau	Starter blend1	BR120; M57_num652;prep1_isolate	4.97	D	D = M57_num652
Muller_prep1_8dd	Muller-Thurgau	Starter blend1	BR120; M57_num652;prep1_isolate	4.97	C	C = 02_MF/03_MF
					D	D = M57_num652
Muller_prep1_EF	Muller-Thurgau	Starter blend1	BR120; M57_num652;prep1_isolate	4.97	D	D = M57_num652
SauvignonMust	Sauvignon	none	02_MF; 03_MF	2.16	C	C = 02_MF/03_MF
Sauvignon_Pdc_4dd	Sauvignon	*Pied de cuve*	02_MF; 03_MF	1.54	A	A = 02_MF/03_MF
Sauvignon_Pdc_EF	Sauvignon	*Pied de cuve*	02_MF; 03_MF	2.16	A	A = 02_MF/03_MF
					B	B = 02_MF/03_MF
Sauvignon_prep1_4dd	Sauvignon	Starter prep1	BR120; M57_num652;prep1_isolate	4.97	D	D = M57_num652
Sauvignon_prep1_EF	Sauvignon	Starter prep1	BR120; M57_num652;prep1_isolate	4.97*	D	D = M57_num652
					F	F = M57_num652
Sauvignon_prep2_4dd	Sauvignon	Starter prep2	prep2_isolate	5.57	G	G = prep2_isolate
Sauvignon_prep2_EF	Sauvignon	Starter prep2	prep2_isolate	4.02*	G	G = prep2_isolate
					E	G = prep2_isolate
ChardonnayMust	Chardonnay	none	02_MF; 03_MF	2.16	A	A = 02_MF/03_MF
					B	B = 02_MF/03_MF
					C	C = 02_MF/03_MF
SolarisMust	Solaris	none	02_MF; 03_MF	2.16	A	A = 02_MF/03_MF
					B	B = 02_MF/03_MF
TraminerMust	Traminer	none	02_MF; 03_MF	2.16	A	A = 02_MF/03_MF
					B	B = 02_MF/03_MF

^1^4dd, 8dd, EF = samples were collected 4 and 8 days after the inoculum and at the End of Fermentation, respectively. The strains present in the samples were identified by means of microsatellites amplification on the total extracted DNA followed by analysis with *SID* (*SID* strains). In addition, strains were isolated from samples and characterized by means of delta amplification, and the band patterns were compared to assess the number and type of strains present (delta amplif. strains). ^1^GLMerror was calculated as the percentage of alleles differing between the predicted profile and the query sample on the total number of alleles in the predicted profile (equation 3 in materials and methods). All the predicted profiles were composed by a total of 324 alleles, with the exception of these annotated with *, which encompassed 325 alleles. ^2^According to the microsatellites profiles, as shown in Supplementary Figure S4.

On the other hand, in both the Muller-Thurgau and the Sauvignon must samples inoculated with the preparation 1 (prep1), three strains were identified since the first sampling after the inoculum, namely BR120, M57_num652, and the strain isolated from the preparation (prep1_isolate), not identified in the *pied de cuve*-inoculated musts. Similarly, in the Sauvignon musts inoculated with prep2 only the inoculated strain (prep2_isolate) was identified. The GLMerror (see equation 3) calculated on must samples ranged from 1.54% to 5.57% (Table 3), and was higher than the GLMerror calculated in previous analyses. The major difference among the analysis on the must samples and on the *in silico* and synthetic samples is that the latter are composed by strains present in the reference dataset (the collection of *S*. *cerevisiae* strains). Thus, the higher error rate calculated for must samples can be ascribed to the absence in the reference dataset of strains present in must samples and could be reduced by expanding the number and genetic variability of the strains composing the reference dataset. This hypothesis is supported by the fact that the predicted profiles of three must samples (Pdc, Sau_prep1_EF and Sau_prep2_EF) encompassed one additional allele not present in the other predicted profiles (* in Table 3), indicating that these samples bore at least one strain not present in the reference dataset. Aiming at the confirmation of the results we obtained on must samples by means of penalized GLM analysis, we used an independent approach to compare the strains isolated from the samples, namely delta amplification (Supplementary Figure S3). Delta amplification showed that fresh musts and samples inoculated with the *pied de cuve*, in which GLM analysis identified two strains (02_MF and 03_MF) were dominated by three common strains, named A, B, and C (Table 3). Generally, the number of strains identified through delta amplification was lower than the number of strains identified by means of GLM analysis, possibly because of the isolation procedure. To assess whether the isolates corresponded to the strains identified by means of penalized GLM analysis, we obtained their microsatellite profiles. Notably, the different strains identified by means of delta amplification showed different microsatellite profiles, indicating that the microsatellite profiling has at least the same discrimination ability than the delta amplification approach. The neighbor joining clustering based on the chord distance among microsatellites profiles of the must isolates and the strains composing our reference dataset clearly revealed a correspondence (*i*.*e*. high similarity) of the must isolates A, B and C with the strains 02_MF and 03_MF, of isolates F and D with the M57_num652 strain and of must isolates E and G with the prep2_isolate strain (Table 3 and Supplementary Figure S4). None of the isolates corresponded to the strains BR120 and prep1_isolate, identified as present in the musts by mean of penalized GLM analysis. Notably, the microsatellite profiles of the A, B, and C isolates were highly similar to the profiles of the 02_MF and 03_MF reference strains, with the A strain being the most similar and the B and C forming a separate sub-cluster (Supplementary Figure S4). Considering the high similarity of the profiles of these strains, it is likely that they are the result of a clonal expansion, which could be evaluated by further investigations (i.e. whole genome sequencing). Although the industrial strains were supposed to dominate the fermentation (because they were selected with this aim and they were inoculated in the musts at high concentrations), the prep1_isolate could no be found by means of isolation followed by delta amplification and yet it was identified by means of penalized GLM analysis, suggesting that it was present in the must, but at low relative abundance. This result further supported the higher sensitivity of our approach compared to the isolation-based one. Furthermore, for samples inoculated with the industrial preparations, the isolation-based analysis generally identified a lower number of strains compared to GLM analysis. This discrepancy could be either ascribed to the inability to isolate strains present at low abundances, or to the incompleteness of the reference dataset used for GLM analysis (suggested by the relatively high GLMerror). The former hypothesis, affecting the delta amplification approach, is strongly supported by the fact that, at any sampling time, we could not find the prep1_isolate in musts inoculated with the prep1. In addition, this hypothesis is further supported by the fact that the samples inoculated with either prep1 or prep2 were shown to encompass a higher number of strains at the second sampling time compared to the first sampling (4dd = first sampling in Table 3). Because no further strains were added during the fermentation process, it is not possible that the number of strains present in the must increases. Rather, the increase of the number of identified strains highlights a deficit in sampling the real biodiversity of the sample. On the other hand, the relatively high GLMerror calculated for these samples (Table 3) supports the hypothesis that the reference dataset used for GLM analysis is incomplete. As a whole, despite we are aware that the reference dataset is far from including the complete biodiversity of *S*. *cerevisiae*, the known weakness of isolation-based approaches, also reported by our aforementioned results, strongly support the first hypothesis.

These results indicate that: (*i*) not every commercial strains overtake the indigenous *S*. *cerevisiae* population, as previously observed^36,37^, but some may remain in traces in the must (i.e. the prep1_isolate, which cannot be detected by means of isolation) and (*ii*) differently from what observed in other studies^38^, the commercial strains used in previous vintages do not persist in the cellar environment nor colonize spontaneous fermentations.

## Conclusions

We propose a method to characterize at the strain level samples containing complex mixtures of *S*. *cerevisiae*. The proposed method was evaluated on *in silico* data and on pools of *S*. *cerevisiae* strains. Both tests supported the possibility to use SSR meta-profile to explore the complexity of *S*. *cerevisiae* populations. The low E_a_ shown by the tested microsatellite loci legitimate the application of this approach on multiple sets of strains. The D_a_ of the single microsatellite loci was considerably low, especially when the number of pooled strains was high. Despite this, the combined use of all the 12 loci allowed the correct identification of pooled strains by mean of lasso analysis, and even strains showing high genetic similarity were discriminated.

The method allowed the dissection of the composition of the *S*. *cerevisiae* population present in grape must fermentations. Thanks to the use of the SSR meta-profile approach we were able to compare the *S*. *cerevisiae* populations in different must fermentations and to assess the fitness of indigenous and commercial strains. Even if the microsatellites profiles of the strains present in the samples were not known a priori as in real samples, this approach was useful to understand whether and how the *S*. *cerevisiae* population changed during the fermentation, and in observing the effects of environmental changes (also encompassing the introduction of external strains) on the composition of the populations. One of the most promising applications of this new method in the winemaking process is the decision of the best inoculum to be used to start the fermentation. Compared to the classical microbial protocols adopted to identify *S*. *cerevisiae* individuals in environmental samples, relying on the isolation, identification and typing of several colonies per samples^9,12–15^, the proposed method is much more rapid. Hence, by applying our method, winemakers will be aware of the composition of the natural *S*. *cerevisiae* population present either in fresh musts or in the produced *pied de cuve*. This information will be fundamental in assisting the decision of either using the cellar-specific *pied de cuve* or facilitate the fermentation by inoculating commercial strains. Our approach may also have applications in environmental and clinical studies, where different yeast strains have been hypothesized to have different outcomes on the host health. However, this variability of the impact of different strains on the host has not yet been shown in epidemiological studies, partially due to the lack of a reliable and rapid method of typing that can be applied in the case of complex mixtures of different strains. Our tool could find a fundamental application in these situations, allowing the identification of strains potentially threatening host health. Furthermore, a recent study compared the effectiveness of Microsatellite Length Polymorphism typing (MLP) as an alternative to Multi Locus Sequence Typing (MLST) for identification of *Candida* spp. strains^39^, showing that the former constitutes a viable alternative to the latter in certain applications. Upon the availability of an extensive strain collection described by MLP, our tool could be extended to this yeast pathogenic species.

## Methods

### SSRs characterization

SSRs lengths were studied at 12 loci: C3, C4, C5, C6, C8, C11, SCYOR267C, SCAAT1, SCAAT3, SCAAT5, YKL172W and YPL9^28^. The primers used to characterize the 12 microsatellite loci are listed in Supplementary Table S3. The PCR mixture consisted of buffer (10x), 2 mM MgCl_2_, 0.1 mM dNTP, 0.32 mM forward primer, 0.32 mM reverse primer, 0.02 U AmpliTaqGold DNA Polymerase (Life Technologies), 25 ng DNA template, water to a final volume of 12.5 microliters. The PCR program consisted of an initial step at 95 °C for 5 minutes, followed by 35 cycles of 95 °C for 0.5 minutes, 57 °C for 2 minutes and 72 °C for 1 minute, and a final elongation step at 60 °C for 30 minutes. Thereafter samples were cooled down to 8 °C until further use. The PCR amplicon sizes of the 12 loci were assessed by capillary electrophoresis using polyacrylamide gels run on a 96-capillary 3730xl DNA Analyzer (Applied Biosystems). Fragment size data were recorded by software GeneMapper (Applied Biosystems) and manually checked. The fragment with the highest fluorescent intensity was scored when SSR-primed products showed band stuttering.

### SSRs meta-profiling performance evaluation

With the term SRRs meta-profiling we indicate the SSR patterns obtained either from samples composed by more than one strain or from environmental samples. The SSR patterns obtained experimentally from the pooled strains (called “pool profile”) were compared to the expected SSR meta-patterns (called “expected profile”) inferred by combining the SSR patterns of the strains composing the pool. The profiles of the single pooled strains were also inspected to identify the presence of strain-specific alleles, namely these alleles that allowed the identification of a strain in a given known pool of strains. The performance of each SSR locus in the analysis on pooled strains was evaluated in terms of discrimination ability D_a_ (equation 1), and average locus error E_a_ (equation 2).

1 $${D}_{a}=\frac{{S}_{sa}}{{S}_{p}}$$

where *S*_sa_ is the number of strains with strain-specific alleles in the pool and *S*_p_ is the number of pooled strains.

2 $${E}_{a}=100\,\ast \,(\frac{1}{{N}_{pool}}\sum \frac{d{a}_{pool}}{ep{a}_{pool}})$$

where *N*_*pool*_ is the number of pooled profiles, *da*_*pool*_ is the number of alleles differing between the given pool and the expected profiles, and *epa*_*pool*_ is the number of alleles in the profile expected for the given pool.

### GLM analysis

Generalized linear model (GLM) analysis with the lasso^34^ penalization was applied to identify the individuals composing complex samples. We refer to individuals composing the population as “parental”, to the reference dataset, composed by individuals’ profiles, as “reference” and to the sample to be analysed as “query”. The reference dataset encompassed the SSR profiles of 274 *S*. *cerevisiae* strains originating from laboratory, grape skins, musts, and fruits (further details in the “Reference collection of *Saccharomyces cerevisiae* strains” section). The analysis was carried out as follows. We initially converted reference’s and query’s SSR alleles into presence/absence profiles. Then we prepared a generalized linear model by using the reference as observations and the query as response variable. The model was trained by the glmnet function of the glmnet R package^40^. We set alpha = 1 (lasso penalty), intercept = F, family = “binomial” and lower . limits = 0 (the latter parameter limits the search space to non-negative coefficients). To avoid overfitting, the regularization parameter lambda minimizing the mean-squared error estimated by cross-validation was chosen. The strains whose individual profiles had non-zero coefficients in the model were tagged as present in the sample. To assess the accuracy of the prediction, we calculated the GLMerror as follows. Once the strains were predicted, we combined their individual (reference) profiles, obtaining a predicted (pooled) profile, which was compared to the observed environmental profile (query). The GLMerror was calculated as the percentage of predicted alleles differing between the predicted and query profiles:

3 $$GLMerror=100\,\ast \,\frac{da}{ep{a}_{pred}}$$

where *da* is the number of alleles differing between the predicted and the query profiles and *epa*_*pred*_ is the number of alleles in the predicted profile.

### Reference collection of Saccharomyces cerevisiae strains

A collection initially composed by 292 environmental and laboratory strains (described in Supplementary Table S4) was used both to generate the *in silico* and synthetic pools and as a reference for the GLM. Two strains isolated from the two preparations used for must inoculation were also added to the reference collection (named prep1_isolate and prep2_isolate). Genomic DNA was extracted by phenol-chloroform-isoamyl alcohol method from single-strain pure cultures and the SSR pattern composed by the amplicon lengths at every analysed locus was determined for each strain. The Chord distance (Dc)^41^, considered the most suitable metric for microsatellite data analyses^42^, was calculated among each couple of SRR patterns using a custom R script. The Neighbor-joining phylogenetic tree was then calculated from the distance matrix using the Phylip Neighbor 3.67 package^43^ and drawn using Figtree (http://tree.bio.ed.ac.uk/). The tree was rooted using the midpoint method. To generate the reference dataset, the strain collection was pruned for redundancy using a recursive procedure. Briefly, GLM analysis was carried out on the complete list of profiles obtained from the strain collection (292 strains) using as query sample each single strain profile separately. In case the analysis identified more than one reference strain associated to a single query profile, only one strain from the list of identified profiles was maintained in the reference dataset. This approach was adopted both to remove the identical strains and to reduce the effect due to the presence in the reference dataset of both haploid and diploid strains. Indeed, diploid strains could be identified by the model as composed by either the exact diploid strain, other haploid strains, or all of them. The data pruning reduced the reference dataset to 274 strains.

### Synthetic pools of *Saccharomyces cerevisiae* strains

Synthetic pools of strains were generated by pooling DNAs of selected *S*. *cerevisiae* isolates and characterizing the SSRs loci in the resulting sample. The set of *S*. *cerevisiae* strains to be pooled was selected according to these requirements: (*i*) the strains were isolated from the same source (to mimic the real samples), (*ii*) the strains bore both strain-specific and shared alleles, (*iii*) the strains showed different levels of genetic similarity (from almost identical strains to divergent strains). Using these criteria, we selected five strains isolated from faeces. To generate the pool, equal amounts of DNAs extracted from pure cultures of the selected five *S*. *cerevisiae* strains were pooled to obtain combinations of strains encompassing from two to five strain DNAs, as described in Supplementary Table S1.

### *In silico* patterns and assessment of the analytical procedures

*In silico* patterns were computationally generated to mimic real samples. Single strains microsatellite patterns were obtained for a collection composed by environmental and laboratory strains as described in the above section. To generate the *in silico* patterns, from 2 to 6 single strain SSR patterns were randomly combined. One thousand *in silico* pattern were generated for each set of strains (2, 3, 4, 5, 6 randomly pooled strain profiles). We thereafter refer to the patterns used to generate the *in silico* profiles as “parental”. The *in silico* patterns were used as query to evaluate the performances of the penalized GLM (see the GLM section for further details on the procedure), using the following parameters: (*i*) True positive rate (equation 4); (*ii*) False positives (the number of strains predicted as composing the pool but not used as parentals); (*iii*) the GLMerror as described in the GLM analysis section (see equation 3) in GLM analysis section).

4 $$True\,positive\,rate=\frac{{P}_{p}}{{Q}_{p}}\,\ast \,{\rm{100}}$$

where *P*_*p*_ is the number parental strains correctly identified in the query and *Q*_*p*_ is the number of parental strains used to generate the *in silico* pattern (query).

### Must samples

Grape musts and different type of ferments were analyzed for different cultivars, namely Sauvignon (Sauvignon blanc), Muller (Muller-Thurgau), Solaris and Chardonnay (Cabernet Chardonnay). Fifty liters of Sauvignon and Muller Thurgau fresh musts were inoculated each with different inocula: (*i*) prep1 or (*ii*) prep2, two preparations of *S*. *cerevisiae* strains selected for white must fermentation, or (*iii*) *pied de cuve* (enrichment of the natural population present on grapes obtained in the winery by fermenting early-harvested grapes). Samples were collected before the inoculum (“must”) and, after the inoculum, every 4 days until the end of fermentation and stored at −80 °C until DNA extraction. Extraction of DNA was carried out from 2 ml thawed must as previously described^3^. The composition of fungal populations was initially explored at the species level by mean of PCR-RFLP on the ITS1-5.8S-ITS2 region. The ITS1-5.8S-ITS2 region was amplified with the primers ITS1: 5′-GTTTCCGTAGGTGAACCTGC-3′ and ITS4: 5′-TCCTCCGCTTATTGATATGC-3′ as previously described^3^. The amplified DNA was then digested with *HaeIII* restriction enzyme as previously described^44^. The obtained band patterns were visualized by mean of gel electrophoresis and analysed by using the free software gelAnalyser (http://www.gelanalyzer.com). SSRs loci were analyzed from DNAs extracted from musts as described above and analysed with GLM.

### Isolation of strains from must and identification via delta elements amplification

One ml of must or ferment samples was plated onto solid YPD (1% Yeast Extract, 2% Peptone, 2% glucose, 2% agar) supplemented with penicillin/streptomycin (10 Units/ml penicillin, 0.01 mg/ml streptomycin). After 3 days incubation at 30 °C, colonies were further isolated and the yeast species was identified by means of PCR-RFLP on the ITS1-5.8S-ITS2 region (see above). *S*. *cerevisiae* isolates were characterized by means of delta element amplification as previously described^45^. The primers d1 (5′-CAAAATTCACCTATWTCTCA-3′) and d2 (5′-GTGGATTTTTATTCCAACA-3′) were used. Delta PCRs were set up from a very small amount of pure yeast colony in 25 microliters of water and 25 microliters of 10 mg/ml of lyticase in Sorbitol 1 M, digested for 30 minutes at 37 °C. After centrifugation, the pellets were treated at 95 °C to inactivate the lyticase and then used as the PCR template. The PCR mixture consisted in buffer (10x, containing 1.5 mM Mg at 1x), 0.25 mM dNTP, 0.5 mM forward primer, 0.5 mM reverse primer, 0.02 U KAPA BioSystems DNA Polymerase (KAPA), water to a final volume of 20 microliters. The PCR thermal program of Delta amplification consisted in: after initial denaturation to activate Taq polymerase at 95 °C for 3 minutes, 95 °C for 30 seconds to denaturate DNA, then 42 °C for 30 seconds (for the first four cycles) and 45 °C for 30 s (for the 30 other cycles) for the annealing cycles and 72 °C for 2 minutes for the extension reaction. The amplification products were analyzed by means of gel electrophoresis (1.5% EtBr 1.5% agarose) in TAE buffer. Amplicon lengths were quantified in comparison to a molecular ladder (FastRuler Middle Range DNA ladder, Thermo Fisher) by using the GelAnalyzer2010a software (freeware). Bands were considered identical when their size deviated by less than 5% of the average size of the group of similar bands. Eventually, strains present in different samples were visually identified by comparing the obtained band patterns.

## Electronic supplementary material

Supplementary materials

Supplementary Table S4
