# Supplementary material for: SaccharomycesIDentifier, SID: strain-level analysis of Saccharomyces cerevisiae populations by using microsatellite meta-patterns
Source: Sci Rep. 2017 Nov 10;7:15343. doi: 10.1038/s41598-017-15729-3 (PMC5681646; doi:10.1038/s41598-017-15729-3)

Supplementary materials

for

*Saccharomyces*IDentifier, *S*ID: strain level analysis of *Saccharomyces cerevisiae* populations by using microsatellite meta-patterns

Irene Stefanini, Davide Albanese, Maddalena Sordo, Jean-Luc Legras, Carlotta De Filippo, Duccio Cavalieri, Claudio Donati

Supplementary Tables

Supplementary Table S1: Composition of synthetic pools of *Saccharomyces cerevisiae* isolates' DNA.

Supplementary Table S2: list of analyzed must samples.

Supplementary Table S3: list of primers used in this study.

Supplementary Figures

Supplementary Figure S1: Cavalli-Sforza distances among the microsatellite profiles of strains selected to be pooled to generate the synthetic pools.

Supplementary Figure S2: PCR-RFLP profiles of must samples.

Supplementary Figure S3: delta amplification analysis on *S. cerevisiae* strains isolated from must samples.

Supplementary Figure S4: Neighbour joining three drawn according to the Cavalli-Sforza distances among the microsatellite profiles of strains composing the reference collection and the strains isolated from the must samples

Other Supplementary Files associated to this study:

Supplementary_Table_S4.xls: list of strains composing the reference dataset. Details on the isolation source and origin are given, as also the SSR length for each analyzed locus.

Supplementary Table S1: Composition of synthetic pools of *Saccharomyces cerevisiae* isolates' DNA. Equal amounts of DNA of the fungal isolates 01_MF, 02_MF, 03_MF, 08_M and, 12_MF were pooled to obtain synthetic mixtures of strains.

**Pool ID**	**Combined strains**
A	01_MF+02_MF
B	02_MF+03_MF
C	01_MF+02_MF+13_MF
D	01_MF+03_MF+13_MF
E	01_MF+02_MF+03_MF+13_MF
F	01_MF+02_MF+03_MF+08_MF+13_MF

Supplementary Table S2: list of analyzed must samples. Details are given on the type of inoculum and on the time of sampling. EF= End of Fermentation.

Sample ID	Cultivar/sample type	inoculum	Time (days)
ChardonnayMust	Chardonnay	-	0
MullerMust	Muller-Thurgau	-	0
SauvignonMust	Sauvignon	-	0
SolarisMust	Solaris	-	0
TraminerMust	Traminer	-	0
Pied de cuve (Pdc)	inoculum	-	
prep1_isolate	inoculum	-	
prep1_isolate	inoculum	-	
Muller_Pdc_4dd	Muller-Thurgau	Pdc	4
Muller_Pdc_8dd	Muller-Thurgau	Pdc	8
Muller_Pdc_EF	Muller-Thurgau	Pdc	12
Muller_prep1_4dd	Muller-Thurgau	Commercial preparation #1	4
Muller_blend1_8dd	Muller-Thurgau	Commercial preparation #1	8
Muller_blend1_EF	Muller-Thurgau	Commercial preparation #1	12
Sauvignon_Pdc_4dd	Sauvignon	Pdc	4
Sauvignon_Pdc_EF	Sauvignon	Pdc	8
Sauvignon_prep1_4dd	Sauvignon	Commercial preparation #1	4
Sauvignon_prep1_EF	Sauvignon	Commercial preparation #1	8
Sauvignon_prep2_4dd	Sauvignon	Commercial preparation #2	4
Sauvignon_prep2_EF	Sauvignon	Commercial preparation #2	8

Supplementary Table S3: list of primers used in this study to sequence the SSR loci. The selection of loci and the design of primers were described by Legras et al. (2005)

**Locus**		**Sequence (5’ → 3’)**
C3	FW	CTTTTTATTTACGAGCGGGCCAT
	RV	AAATCTCATGCCTGTGAGGGGTAT
C4	FW	AGGAGAAAAATGCTGTTTATTCTGACC
	RV	TTTTCCTCCGGGACGTGAAATA
C5	FW	TGACACAATAGCAATGGCCTTCA
	RV	GCAAGCGACTAGAACAACAATCACA
C6	FW	GTGGCATCATATCTGTCAATTTTATCAC
	RV	CAATCAAGCAAAAGATCGGCCT
C8	FW	CAGGTCGTTCTAACGTTGGTAAAATG
	RV	GCTGTTGCTGTTGGTAGCATTACTGT
C11	FW	TTCCATCATAACCGTCTGGGATT
	RV	TGCCTTTTTCTTAGATGGGCTTTC
SCYOR267C	FW	TACTAACGTCAACACTGCTGCCAA
	RV	GGATCTACTTGCAGTATACGGG
SCAAT1	FW	AAAGCGTAAGCAATGGTGTAGATACTT
	RV	CAAGCCTCTTCAAGCATGACCTTT
SCAAT3	FW	TGGGAGGAGGGAAATGGACAG
	RV	TTCAGTTACCCGCACAATCTA
SCAAT5	FW	AGCATAATTGGAGGCAGTAAAGCA
	RV	TCTCCGTCTTTTTTGTACTGCGTG
YKL172W	FW	CAGGACGCTACCGAAGCTCAAAAG
	RV	ACTTTTGGCCAATTTCTCAAGAT
YPL9	FW	AACCCATTGACCTCGTTACTATCGT
	RV	TTCGATGGCTCTGATAACTCCATTC

Supplementary Figure S1: Cavalli-Sforza distances among the strains selected to be pooled to generate the synthetic pools.

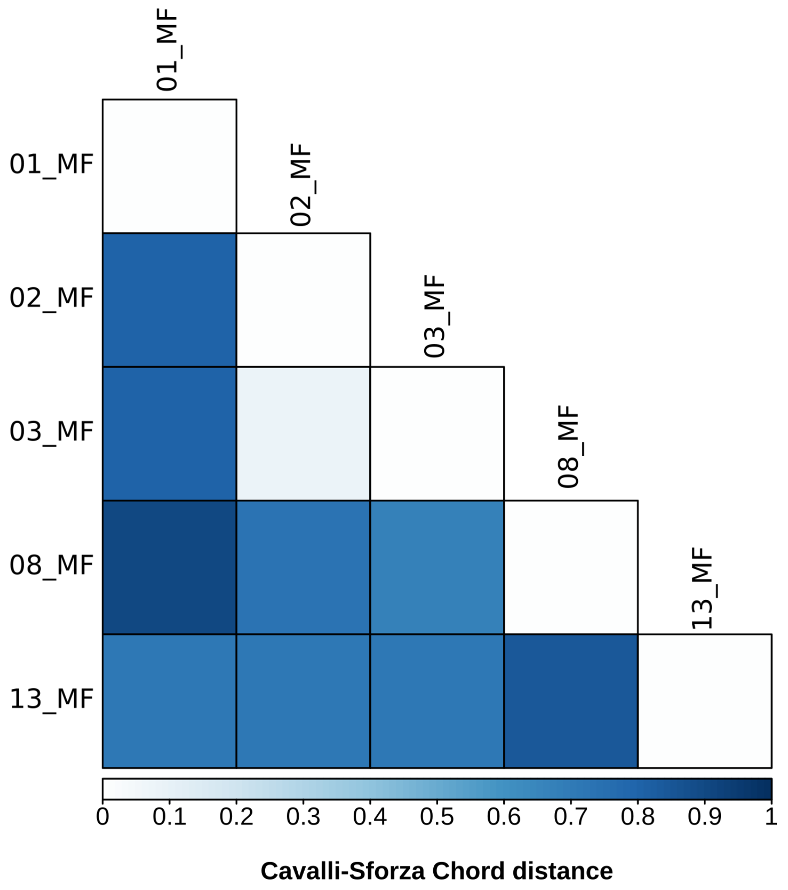

Supplementary Figure S2: PCR-RFLP profiles of must samples. The ITS1-5.8-ITS2 region was amplified and the amplified fragment was digested with the *HaeIII* restriction enzyme. Not-labeled profiles are relative to conditions not analyzed in this study.

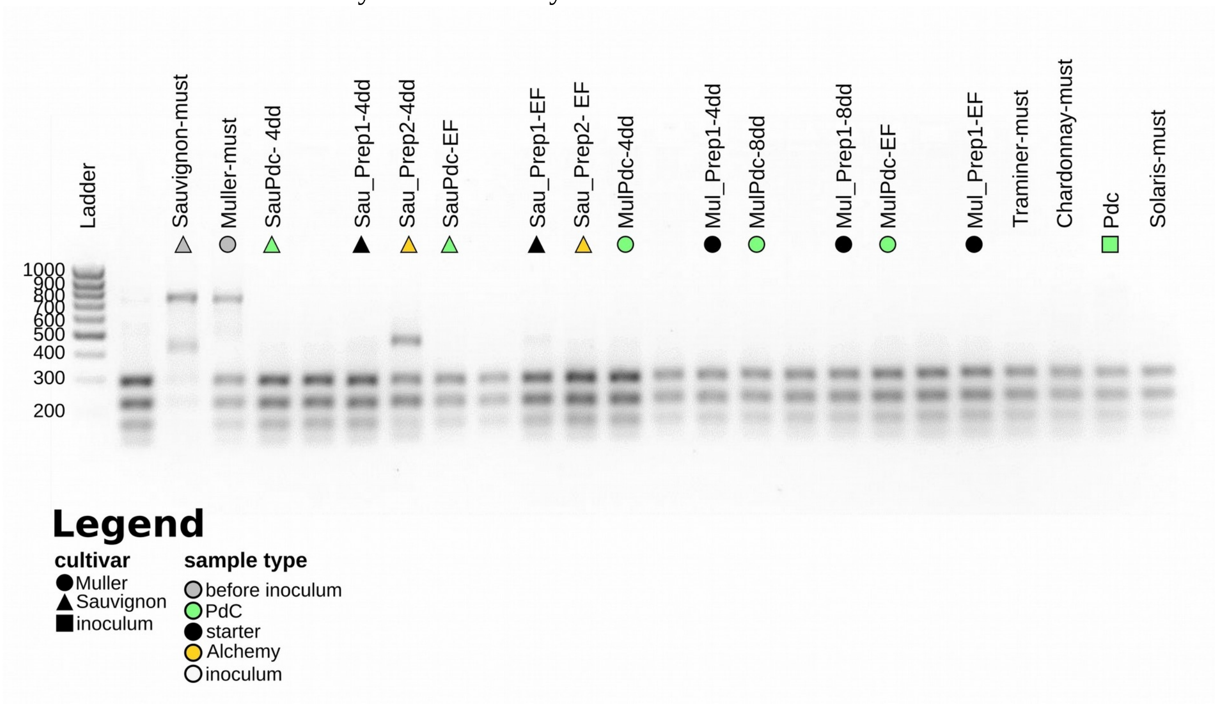

Supplementary Figure S3: delta amplification analysis on *S. cerevisiae* isolated from must samples. **a-** amplificates were analysed by means of gel electrophoresis, **b-** the band profiles were compared among strains to evaluate the composition of the sample yeast populations.

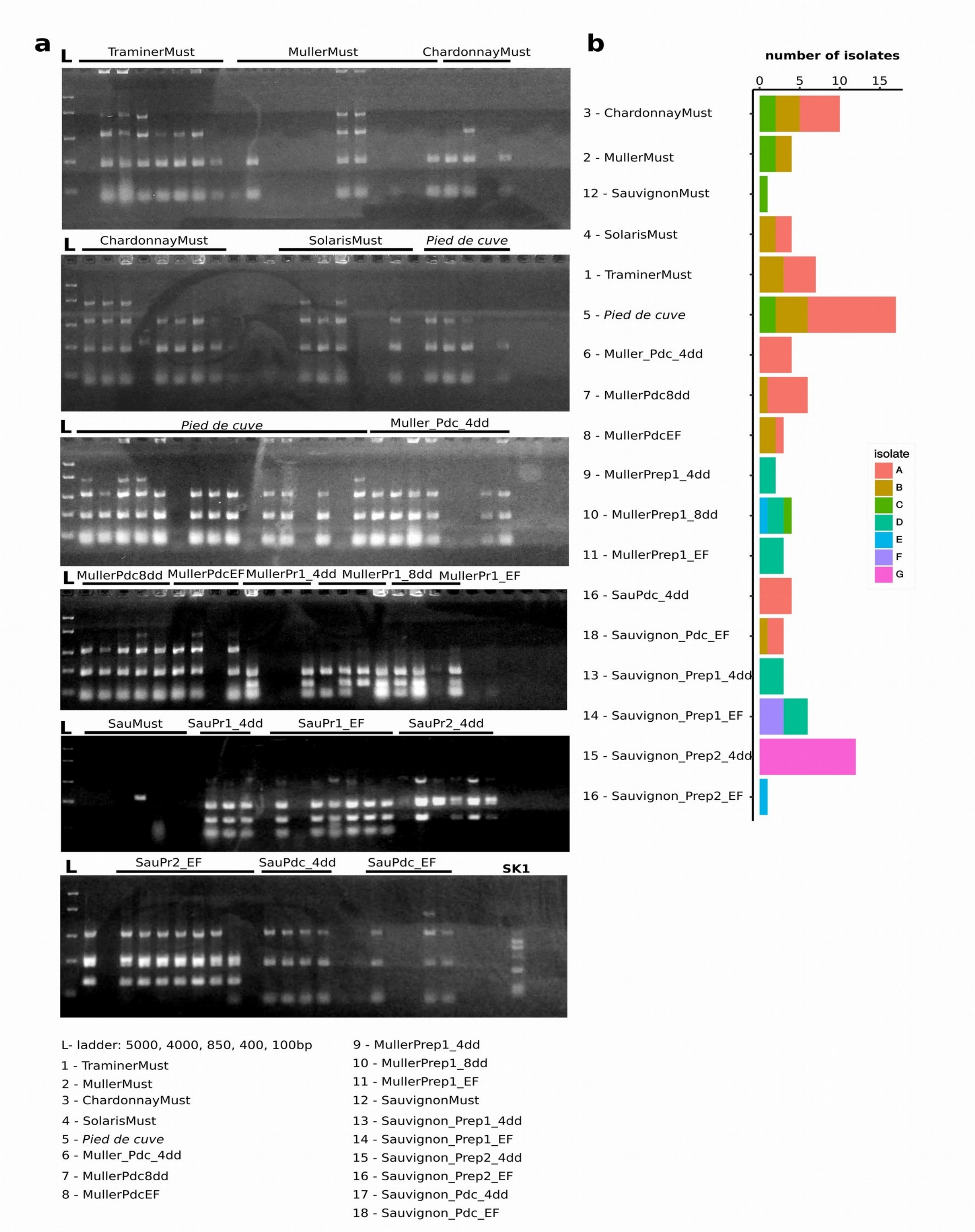

Supplementary Figure S4: Neighbour joining three drawn according to the Cavalli-Sforza distances among the microsatellite profiles of strains composing the reference collection and the strains isolated from the must samples. blue= strains isolated from must samples; red= strains identified by mean of the lasso analysis.

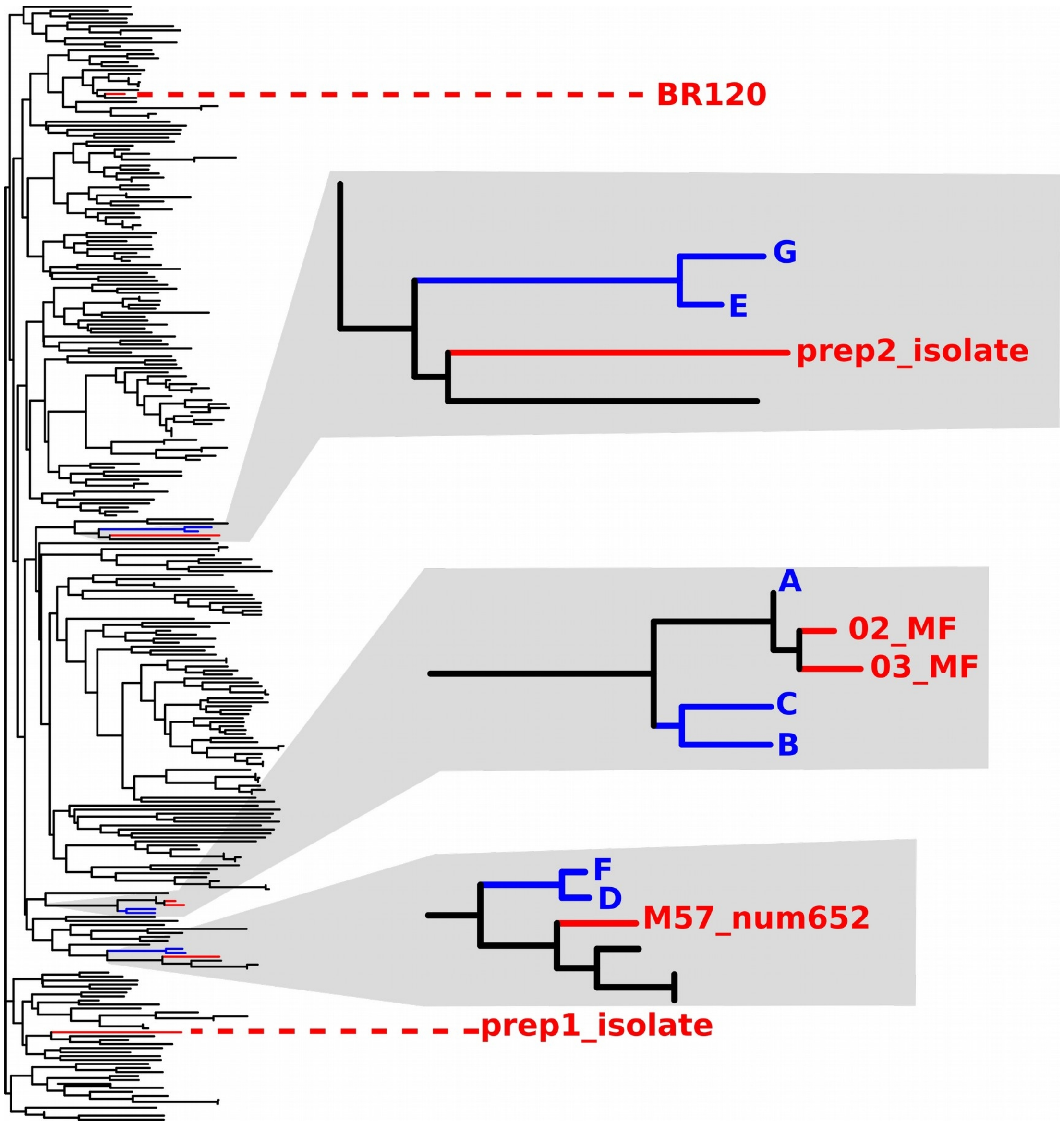

Supplement: Supplementary file 1 — Supplementary materials [file 41598_2017_15729_MOESM1_ESM.pdf]
